# Monitoring vascular normalization: new opportunities for mitochondrial inhibitors in breast cancer

**DOI:** 10.18632/oncoscience.523

**Published:** 2021-02-25

**Authors:** Silvana Mouron, Maria J. Bueno, Manuel Muñoz, Miguel Quintela-Fandino

**Affiliations:** ^1^Breast Cancer Clinical Research Unit - Clinical Research Program, CNIO - Spanish National Cancer Research Center, Madrid, Spain; ^2^Medical Oncology, Hospital Universitario de Fuenlabrada, Fuenlabrada, Madrid, Spain; ^3^Medical Oncology, Hospital Universitario Quiron, Pozuelo de Alarcon, Madrid, Spain; ^4^Department of Medicine, Universidad Autonóma de Madrid, Madrid, Spain

**Keywords:** breast cancer, clinical trial, antiangiogenics, mitochondrial inhibitors

## Abstract

Preclinical evidence indicates the potential of targeting mitochondrial respiration as a therapeutic strategy. We previously demonstrated that mitochondrial inhibitors’ efficacy was restricted to a metabolic context in which mitochondrial respiration was the predominant energy source, a situation achievable by inducing vascular normalization/hypoxia correction with antiangiogenics. Using molecular imaging, we showed how the same antiangiogenic agent may display different normalizing properties in patients with the same tumor type. This is of key importance, since patients experiencing normalization seem to get more benefit from standard chemotherapy combinations, and also could be eligible for combination with antimitochondrial agents. This scenario emphasizes the need for monitoring vascular normalization in order to optimize the use of antiangiogenics. We have also proposed a method to evaluate anti-mitochondrial agents’ pharmacodynamics; despite promising accuracy in animal studies the clinical results were inconclusive, highlighting the need for research in this field. Regarding patients that respond to antiangiogenics increasing vessel abnormality, in this case an immunosuppressive tumor microenvironment is generated. Whether anti-mitochondrial agents can positively modulate the activity of T effector cell subpopulations remains an area of active research. Our research sheds light on the importance of refining the use of antiangiogenics, highlighting the relevance of tracing vascular normalization as a potential biomarker for antiangiogenics to assist patient-tailored medicine and exploring the role of mitochondrial inhibitors in the context of vascular normalization and correction of hypoxia.

## INTRODUCTION

**Figure 1:**
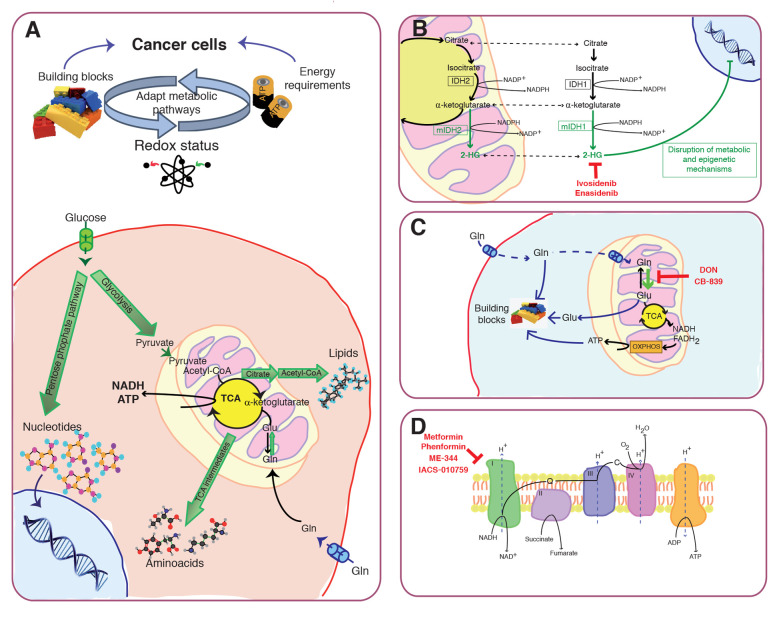
Cancer metabolic remodelling: molecular pathways and potential interventional strategies. **A**. Cancer cells develop metabolic plasticity in order to satisfy the sufficient supply of reduced carbon sources for the generation of ATP, building blocks and reducing power to support cancer cell survival and proliferation. The figure depicts the key features behind the Warburg effect in cancer cells, including glycolysis, TCA cycle, pentose pyruvate pathway, glutamine metabolism, and use of TCA cycle intermediates to synthesize lipids, aminoacid and nucleotides. **B**. Isocitrate dehydrogenases (IDH1 and IDH2) are critical metabolic enzymes that catalyze the oxidative decarboxylation of isocitrate to α-ketoglutarate (αKG), NAD(P)H, and CO2. IDHs epigenetically control gene expression through effects on αKG-dependent dioxygenases, maintain redox balance and promote anaplerosis by providing cells with NADPH and precursor substrates for macromolecular synthesis. Mutant IDH1/2 causes accumulation of the oncometabolite, 2-hydroxyglutarate (2-HG), which has been considered as an early and critical contributor to oncogenesis. So far, two mutant IDH inhibitors, enasidenib and ivosidenib have been approved for IDH-mutant relapsed or refractory acute myeloid leukaemia (AML). **C**. Glutaminolysis is an important energy source in tumor cells. Glutamine (Gln) is an important supplier of building blocks that support macromolecular biosynthesis and antioxidant molecules, and in turn, cell mass accumulation and proliferation. The antitumor effects of several inhibitors of glutaminolysis such as CB-839 and 6-diazo-5-oxy-L-norleucine (DON) have been explored in preclinical studies. **D**. Mitochondria play a central role in cancer development. Targeting electron transport chain complex I using specific inhibitors such as metformin, phenformin, ME-344 and IACS-010759 is considered an attractive therapeutic strategy to selectively kill cancer cells.

Over the last few decades, searching effective therapies for advanced-stage cancers has been the focus of many research studies. The study of cancer-associated metabolic remodelling has emerged as a promising strategy for pharmaceutical intervention in cancer. Cancer cells must develop metabolic plasticity in order to allow them to satisfy the sufficient supply of reduced carbon sources for the generation of ATP, building blocks and reducing power to support and enable rapid proliferation, continuous growth, survival, invasion and metastasis [1] (Figure 1A). Because of the altered metabolic features during tumorigenesis, it is conceivable that cancer cells could develop resistance to inhibition of a particular metabolic pathway by up-regulating compensatory ones. Therefore, targeting multiple metabolic pathways simultaneously or targeting a particular metabolic pathway in combination with therapies against oncogenic or signalling pathways may be exploited as a rational and attractive therapeutic strategy in cancer.

### Metabolic therapies in oncology

In this context, the study of mutations in genes directly implicated in the re-arranging of metabolic pathways and metabolic reprogramming as a mechanism of acquired resistance to different anticancer drugs of some tumors become novel pursued opportunities in the discovery of new agents and optimization of personalized medicine. The identification of isocitrate dehydrogenase (IDH) mutations across multiple cancer types including hematologic malignancies, cholangiocarcinoma and chondrosarcoma revolutionized the potential for targeting these diseases [2]. *IDH1/2* mutations generate an oncometabolite product, 2-hydroxyglutarate (2-HG), which has been linked to the disruption of metabolic and epigenetic mechanisms responsible for cellular differentiation and is likely an early and critical contributor to oncogenesis [3] (Figure 1B). So far, two mutant IDH inhibitors, enasidenib and ivosidenib have been approved by the US Food and Drug Administration (FDA) - for IDH-mutant relapsed or refractory acute myeloid leukaemia (AML) and continue to be studied in trials in hematologic malignancies, as well as in glioma, cholangiocarcinoma, and chondrosarcoma [4-7].

Multiple discoveries have been done linking cancer metabolism and cancer progression. However, the well-known cancer-associated alterations in metabolism, and the key roles of the two main pathways, glycolysis and glutaminolysis, in fostering tumor growth have not been translated into remarkable advances in the clinical practice [8]. To date, the antitumor effects of several inhibitors of glycolysis (ionidamine) and glutaminolysis (CB-839, DON and PEG-PGA) have been explored in preclinical studies [9, 10] (Figure 1C). So far, current clinical trials in advanced refractory solid tumors to block glutaminolysis have shown only modest clinical efficacy and considerable systemic toxicity [10, 11].

### Targeting mitochondrial respiration in cancer

Mitochondria are important cellular organelles that play essential roles in energy metabolism, calcium homeostasis, redox maintenance and apoptosis [12, 13]. It has been demonstrated that mitochondria play a central role in cancer development as well, by contributing to most of the classical hallmarks of cancer, including metabolic re-programming, sustained proliferation, apoptosis resistance, invasion and induction of angiogenesis [14]. Mitochondria are key for almost all facets of tumor progression, not only as a major source of ATP, but also due to their ability to provide building blocks for anabolism via anaplerosis, their capacity to produce reactive oxygen species (ROS) and their central position in regulating cell death signalling [15]. As such, targeting mitochondria using proper pharmacologic agents is considered an attractive therapeutic strategy to selectively kill cancer cells.

Preclinical studies and retrospective population-based studies have suggested antitumor activity of mitochondrial inhibitors, mainly the electron transport chain complex I inhibitors metformin and phenformin, alone or in combination with inhibitors of other oncogenic pathways [16-19]6-19] (Figure 1D). However, a randomized clinical trial with metformin failed to show outcome improvement over gemcitabine and erlotinib alone in advanced pancreatic cancer [20]. Similarly, the addition of metformin to standard treatment for lung cancer patients did not improve overall survival (OS) or the progression-free survival (PFS) [21, 22]. Also, clinical trials from metastatic breast cancer patients failed to show long-term efficacy of metformin plus chemotherapy versus chemotherapy alone [23, 24] or in the metformin plus everolimus arm compared to the control arm [25]. Moreover, metformin´s efficacy as an anticancer agent is dependent on the tumor expression of organic cation transporters (OCTs) [16] what complicates its potential use as widespread therapy for cancer therapy. Phenformin, conversely, does not require expression of OCTs for entering the cancer cell; however, it has been withdrawn from clinical use because of frequent occurrence of lactic acidosis [26, 27]. Recently, novel mitochondrial inhibitors targeting mitochondrial oxidative phosphorylation (OXPHOS) have shown potential antitumoral activity in models of brain cancer, AML and lung adenocarcinomas; [28, 29] and are currently under early-phase evaluation in clinical trials [30]. Moreover, ME-344 is a synthetic small molecule based on the isoflavone ring structure with mitochondrial complex I inhibitory properties [31] that showed good tolerability in a phase I clinical trial of patients with refractory solid tumors [32] (Figure 1D). However, no meaningful activity was observed in a subsequent trial in combination with topotecan (a topoisomerase inhibitor) in locally advanced or metastatic small cell lung (SCLC), ovarian or cervical cancer [33].

### ME-344 and breast cancer

The lack of anticancer activity of metformin or ME-344 alone or in combination in unselected patients could be explained by metabolic reprogramming events that compensate the blocked metabolic pathway with an alternative one to reach the energetic and biosynthetic requirements of tumor cells. In most epithelial malignancies (especially in MAPK- and/or Pi3K-AKT-activated tumors), the energy requirement relies on high glucose uptake and glycolytic metabolism. Nevertheless, we have previously described that breast cancer cells have an impressive metabolic plasticity that is regulated by the heterogeneous tumor microenvironment. The accessibility of nutrients and oxygen influences the metabolism of cancer cells that shift between glycolysis and mitochondrial respiration. This ability to switch from one metabolic source to another causes that pharmacological inhibition with phenformin was not effective when administered monotherapy, since upon blocking the mitochondria tumors become more glycolytic. Taking into account the previous findings, we have worked to find niches in which a mitochondrial inhibitor could be highly effective – namely, situations in which a given drug would render mitochondrial respiration essential.

**Figure 2:**
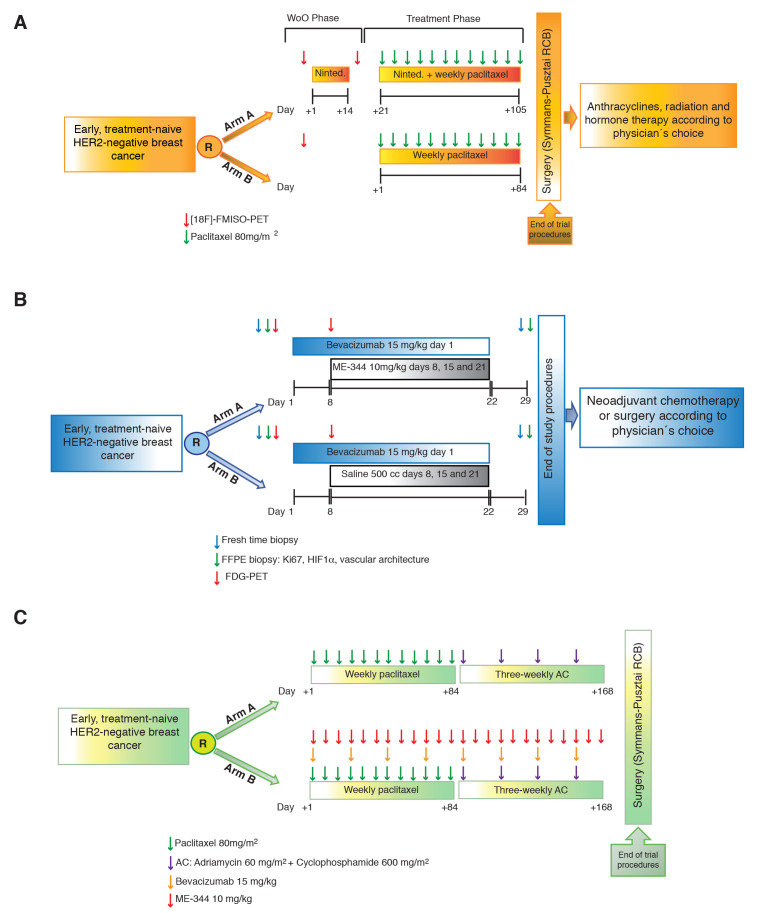
Clinical trials studying vascular normalization and role of mitochondrial inhibitors. **A**. Schematics of the BR-003 trial. Early HER2-negative treatment-naive breast cancer patients were randomized to standard chemotherapy with weekly paclitaxel (standard arm) or the same chemotherapy plus the multikinase inhibitor antiangiogenic agent nintedanib (experimental arm). The experimental arm was preceded by a 14-day window-of-opportunity part of nintedanib monotherapy. A [18F]-FMISO-PET was performed before and after the window-of-opportunity to measure individually the rate of hypoxia correction (or increment) in response to nintedanib. Patients in the standard arm underwent only a baseline 18F-fluoromisonidazole PET. Patients with increased baseline hypoxia responded worse to chemotherapy regardless of the arm. The experimental arm demonstrated that nintedanib was able to correct hypoxia in approximately 1/3 of the patients. **B**. Patients with early HER2-negative treatment-naïve breast cancer were randomized to a single dose of bevacizumab plus placebo or bevacizumab plus the mitochondrial inhibitor ME-344 (BR-009 trial). Patients underwent a FDG-PET before the bevacizumab dose and 8 days after in order to ascertain the effects of bevacizumab. After the second PET, patients started on weekly ME-344 or placebo for three weekly doses. The trial measured the surrogate marker of efficacy decrease of Ki67 replicative fraction. Patients also underwent a tissue biopsy baseline and in day +28. This design allowed answering two questions: 1) whether FDG-PET correlated with histologic changes that monitored vascular normalization such as hypoxia correction and improved vessel architecture; and 2) whether the mitochondrial inhibitor ME-344 increased the biologic activity of bevacizumab in patients in whom bevacizumab caused normalization. We found that FDG-PET accurately detected vascular normalization and that the activity of ME-344 was particularly high upon hypoxia correction and vascular normalization **C**. The design of the BR-009 trial did not measure the effect of ME-344 in pathologic complete response (pCR), the standard endpoint for the adjuvant setting. In order to answer in a definitive manner whether adding a mitochondrial inhibitor upon antiangiogenic-induced vascular normalization improves current rates of pCR in early breast cancer, the design should be similar to the one depicted in this figure: patients would undergo standard chemotherapy alone (standard arm) or in combination with bevacizumab and ME-344 for 24 weeks prior to surgery (the BR-009 trial was only a proof-of-concept 4-week trial that can serve as a justification to run the proposed trial on the basis of the results observed in the Ki67 replicative fraction, a well-accepted surrogate marker of pCR).

Our previous studies suggest that tumors treated with antiangiogenic agents may display a dual pattern of adaptive microenvironmental responses: one induces vascular and stromal normalization coupled with re-oxygenation, whereas the second adaptation is elicited through vascular trimming and increased hypoxia. We have shown in preclinical models of acquired resistance against antiangiogenics in highly-glycolytic tumors that when antiangiogenics induce vascular normalization and hypoxia correction they experience a metabolic shift consisting on suppression of glycolysis and upregulation of mitochondrial respiration [34]. This shift renders mitochondrial metabolism essential for tumor cell survival and mitochondrial inhibitors such as phenformin or ME-344 display synergy with the vascular-normalizing/re-oxygenating agents. We termed this phenomenon “metabolic synthetic lethality” [34]. Hence, the benefit of mitochondrial inhibitors seems to depend on the tumor metabolic context and would mainly exert antitumor effect when mitochondrial respiration is the only available energetic source.

## Antiangiogenics and breast cancer: A potential niche for mitochondrial inhibitors

### Tracing vascular normalization as a potential biomarker for antiangiogenics

In 2008, the antiangiogenic bevacizumab was FDA-approved in combination with paclitaxel for the treatment of metastatic breast cancer. Despite of the transient activity demonstrated by bevacizumab, consisting on an increased response rate and longer progression-free survival (PFS) compared to paclitaxel alone; [35, 36] this combination has not shown a durable clinical benefit based on an overall survival (OS) benefit in breast cancer [37, 38]. Based on these data, in 2011, the FDA withdrew bevacizumab as the treatment for metastatic breast cancer. The inability of bevacizumab to generate significant clinical benefit may be at least, partially explained by the lack of biomarkers to predict efficacy and therefore the possibility of an increased clinical benefit of bevacizumab in a subgroup of patients that has yet to be identified cannot be excluded. We focused our research in trying to refine the use of antiangiogenics, finding potential biomarkers of activity and exploring the role of mitochondrial inhibitors in the context of vascular normalization and correction of hypoxia.

First, we have shown in preclinical models that vascular normalization or increased abnormality of the vasculature in the tumor may be traced with molecular imaging. One hand, we have demonstrated in highly-glycolytic tumors that when antiangiogenics induce vascular and stromal normalization there is a decrease of tumor glucose uptake and increased oxygenation, which translates into lower ^18^F-fluorodeoxyglucose avidity at Positron Emission Tomography (FDG-PET) [34]. On the other hand, ^18^F-fluoromisonidazole-PET ([18F]-FMISO-PET) is able to detect hypoxic areas because of increased vascular abnormality by binding of reduced-positron-emitting nitroimidazole to tissue areas with less than 1% oxygen. We have demonstrated using preclinical models that in response to short-course antiangiogenic treatment, [18F]-FMISO-PET could detect those cases in which interstitial hypoxia is corrected and vascular abnormality is reverted, leading to improved chemotherapy delivery [39]. Interestingly, we have observed that the same cancer model can experience an increase in hypoxia and vessel abnormality in response to an antiangiogenic (monoclonal antibody (mAb) against VEGF) and vascular normalization and tumor re-oxygenation in response to other antiangiogenic agents (nintedanib and dovitinib, multi-tyrosine-kinase inhibitors (TKIs) [34]. In addition, we showed how the same antiangiogenic agent does not necessarily induce a homogeneous normalizing response across different tumors of the same histology (e.g., we have shown that the TKI dovitinib induced normalization of a pancreas cancer model but increased abnormality in a second pancreas cancer model [39], highlighting the relevance of using molecular imaging to trace the occurrence of normalization or increased abnormality of vasculature in an individual basis.

We then tried to validate those results in the context of a randomized neoadjuvant trial in early HER2-negative breast cancer (CNIO-BR-003 trial; Figure 2A). Patients were randomized to standard chemotherapy (standard arm) or chemotherapy plus the multitargeted antiangiogenic nintedanib (experimental arm). Patients in the experimental arm were treated in a window-of-opportunity part with nintedanib monotherapy for two weeks, preceded and followed by a [18F]-FMISO-PET scan, prior to the combination phase [40]. As expected, a heterogeneous re-oxygenation response was observed. Twenty-four per cent of nintedanib-treated patients experienced vascular normalization and reoxygenation, evidenced by a reduction in 18F-FMISO tumor-to-muscle ratio (TMR) uptake. However, the remainder patients mostly stayed stable within the plus- or minus-10% *versus* baseline boundaries in TMR uptake, with the exception of one patient that had increased hypoxia. Patients with tumors more oxygenated at the baseline had better response rates than those with high baseline hypoxia; however, the main readout of this trial is that when tumors of the same type (early HER2-negative tumors) are exposed to a given antiangiogenic (nintedanib), some patients can experience normalization whereas others can experience increased abnormality [40]. In a different trial (BR-009 trial) we also studied the role of FDG-PET scan to detect vascular normalization. This second trial consisted on a randomized window-of-opportunity trial in early HER2-negative breast cancer of bevacizumab monotherapy for 4 weeks *versus* bevacizumab plus the antimitochondrial agent ME-344 (Figure 2B). An FDG-PET scan was scheduled on day 0 and 8 days after the first bevacizumab dose [41]. In this trial, changes in tumor tissue (hypoxia staining with HIF1α and assessment of microvascular architecture) were determined along the window-of-opportunity in order to ascertain the accuracy of the PET scans. Interestingly, approximately 2/3 of the patients experienced a deterioration of the microvascular architecture and tissue oxygenation, accordingly with the PET findings [41]. In both clinical studies, patients that showed antiangiogenic-induced vascular normalization had a higher chance of experiencing clinical benefit than those that do not experienced re-oxygenation and stromal normalization [40, 41], but then again, we believe that the second interesting conclusion is the confirmation of the second preclinical observation: patients with the same tumor type (early HER2-negative tumors) exposed to different antiangiogenics (bevacizumab and nintedanib) experience different rates of hypoxia and vessel abnormality correction.

Antiangiogenic agents were developed against different targets and differ in their molecular structure and in their affinity and K_M_ for the targets; bevacizumab inhibits VEGF-A [42] whereas nintedanib is a multitargeted tyrosine kinase (TKI) inhibitor that blocks several axes involved in the maintenance of an abnormal tumor stroma such as VEGFR1-4, PDGFRα and β, or FGFR1-3 [43]. Therefore, antiangiogenic agent characteristics are important features to take into account at the moment of choice for therapeutic options to ensure the potential clinical benefit and, molecular imaging appears as a useful tool to assist patient-tailored medicine in the identification of antiangiogenic-induced vascular normalization, which seems to be related with improved benefit in the clinical setting.

## ME-344 and antiangiogenic-induced vascular normalization: successful efficacy data and inconclusive pharmacodynamics

As we have shown, the mechanisms of antiangiogenic-acquired resistance involve metabolic reprogramming events consisting on downregulation of glycolysis and upregulation of mitochondrial respiration. In this context, the benefit of mitochondrial inhibitors was enhanced in several preclinical models of breast and lung cancer (both genetically engineered immuno-competent models and immuno-compromised xenograft models). Thus, we aimed to study the concept of metabolic synthetic lethality in the clinical setting. The randomized phase 0 BR-009 trial was designed for that purpose (Figure 2B): treatment-naïve early HER2-negative breast cancer patients received a single bevacizumab dose preceded and followed by FDG-PET to detect vascular normalization, and then were randomized to receive three weekly ME-344 or placebo doses [41]. Despite of the lack of biologic activity and antitumor efficacy of ME-344 observed in a previous clinical trial [33], our results confirmed a greater benefit in those patients that experienced bevacizumab-induced vascular normalization, suggesting a context-dependent effect of mitochondrial inhibitors [41]. The addition of ME-344 in the experimental arm after bevacizumab dosing led to a significant biologic activity, consisting on an average 23.4% decrease in the tumor Ki67 replicative fraction compared to a 186% increase in the placebo arm. Interestingly, the Ki67 replicative fraction decrease was higher in the patients that experienced vascular normalization according to the FDG-PET scan. Approximately 1/3 of the patients experienced vascular normalization following bevacizumab in both arms; however, the Ki67 replicative fraction decreased by an average of 33% in the normalized patients receiving ME-344 (experimental arm), compared to an average increase of 12% in the normalized patients that received placebo after bevacizumab (standard arm) [41]. Taking into account that patients only received a single dose of bevacizumab and three-doses of ME-344, we could hypothesize that a long-term administration of bevacizumab plus ME-344 during the neoadjuvant setting may translate into a better tumor control rate; however a definitive answer in that matter should be addressed with a randomized clinical trial comparing standard chemotherapy *versus* chemotherapy combined with bevacizumab and ME-344, measuring pathologic complete response (pCR) as primary trial outcome (Figure 2C).

**Figure 3:**
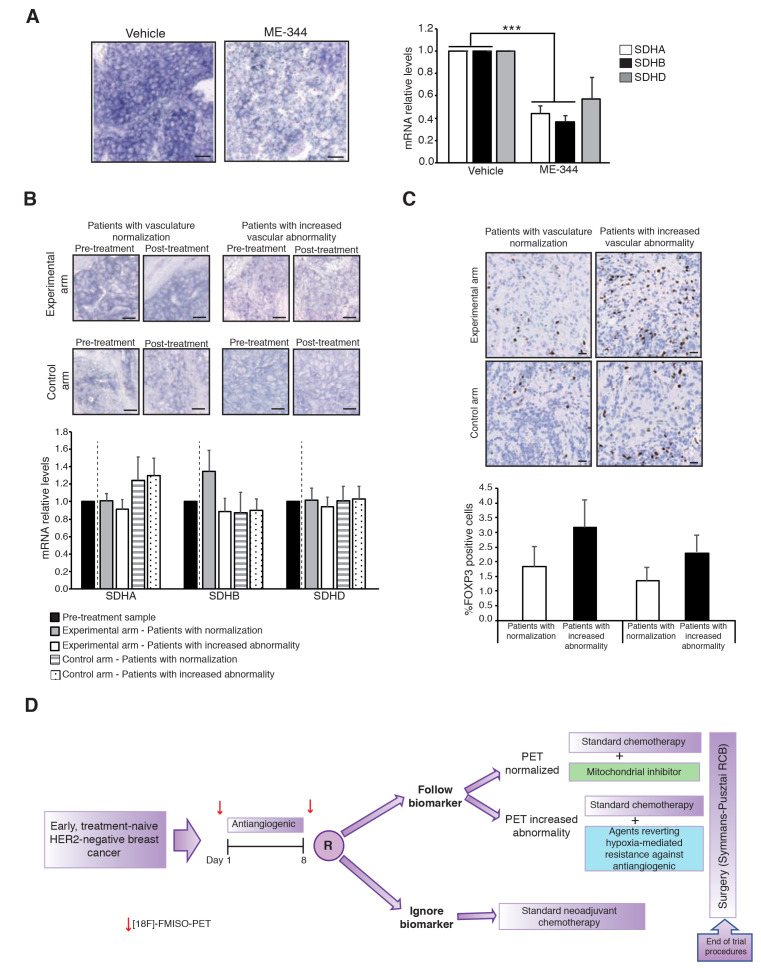
Pharmacodynamics of ME-344. **A**. Left: Representative images from SDH-EHC in PyMT tumors after ME-344 or vehicle treatment. SDH activity was significantly reduced after ME-344 treatment. Scale 20 μM. Right: mRNA levels of three of the components of succinate dehydrogenase complex (SDH A, B and D) in tumors from PyMT breast cancer model (vehicle n=5 and ME-344 n=5 treated mice). SDHA and B were significantly downregulated after ME-344 treatment. Values were normalized relative to β-actin mRNA and presented as mean ±SD. P value *** = p<0.001. **B**. Upper: Representative images from SDH-EHC in patients pre- versus post-ME-344 or placebo treatment. Scale 20 μM. Down: mRNA levels of SDH units in samples from patients of BR009 trial. In each patient post-treatment sample were normalized against pre-treatment tumor and mean values were represented. **C**. FOXP3 staining changes from control (exposed to placebo) and experimental arm (exposed to ME-344) were compared among patients that, according to FDG-PET, experienced vascular normalization in response to the bevacizumab dose and those patients that increase vascular abnormality and hypoxia. We observed a decrease in the percentage of FOXP3+-positive T_regs_ cells in those patients that experienced bevacizumab-vascular normalization compared with those patients with increased abnormality of vasculature in both, the experimental and the control arm. Scale 20 μM.**D**. A potential way to tailor neoadjuvant treatment in breast cancer taking into account the effects of antiangiogenics in cancer metabolism and the tumor stroma is the depicted trial schedule: patients would undergo a window-of-opportunity 14-day monotherapy phase with antiangiogenic treatment preceded and followed by a [18F]-FMISO-PET. The, patients would be randomized to either ignore the PET scan and proceed to routine chemotherapy, or allocate the treatment according to the PET scan results: patients displaying normalization would receive the antiangiogenic agent plus a mitochondrial inhibitor in combination with chemotherapy; conversely, patients experiencing an increase in abnormality would receive chemotherapy plus a combo – yet to be defined by ongoing research – that enhances the efficacy in this context. This trial would allow answering two questions: 1) whether assessing the effects in tumor re-oxygenation and acting consequently to those changes improves treatment outcomes; and 2) whether the proposed combinations for vascular normalization and vascular abnormality actually perform better than standard chemotherapy.

It is also worth noting that, akin any other anticancer drug, the efficacy of neither bevacizumab nor ME-344 is homogeneous across all patients. In all cases, even with drugs targeting oncogenic addiction drivers such as mutant EGFR or BCR-ABL in lung cancer [44] or chronic myeloid leukemia [45], a variable number of patients do not obtain benefit from the treatment. The development of biomarkers is always necessary to refine the use of anticancer drugs. In the case of antiangiogenics, measuring hypoxia by [18F]-FMISO-PET or FDG-PET seem to narrow-down the percentage of patients that experience benefit from them. However, further standardization of quantitation and acquisition methods would be required in order to adopt an imaging test as a predictive factor in a widespread manner. Regarding mitochondrial inhibitors, the field is less advanced. A key feature that could decrease the attrition rate of novel drug candidates is the demonstration of pharmacodynamic activity and target engagement *in vivo* during early phases of clinical development. For example, in preclinical studies we observed that an enzymohistochemical assay (EHC) that measured the activity of succinate dehydrogenase (SDH) (an enzyme shared by the Krebs cycle and the mitochondrial electron transport chain) [46] in fresh tissue was able to distinguish tumors with high mitochondrial activity *versus* those that relied mostly in glycolysis. Interestingly, phenformin or ME-344, that block the activity of mitochondrial complex I, congruently were able to turn negative this enzymohistochemical assay, translating decreased/suppressed mitochondrial respiration [34]. Although the BR-009 trial yielded a positive efficacy outcome, when we attempted to show the *in vivo* pharmacodynamic engagement of ME-344 with this enzymohistochemical assay in patients, we were unable to gather any conclusive result. ME-344 was demonstrated to be a potent inhibitor of respiratory complex I *in vitro* and this inhibition causes an immediate reduction of mitochondrial respiration and loss of the mitochondrial membrane potential with the subsequent destabilization of the OXPHOS complexes [31]. Although it does not exert inhibitory effects over the other complexes during short periods *in vitro*, the blockade of mitochondrial complex I *in vivo* could be potentially compensated by an increased activity of complex II (succinate dehydrogenase) resulting in a significant increase in SDH staining. In our preclinical experiments, we analysed both the expression of the components of succinate dehydrogenase (SDH complex A, B and D) and we performed the enzymohistochemistry test in the breast cancer model PyMT in animals treated with vehicle or ME-344, obtaining the results shown in Figure 3A. The enzymohistochemical staining displayed wide suppression of the respiratory activity, and the transcriptional levels of SDH units A and B were significantly down-regulated. However, in patients, pre- *versus* post- treatment samples in the experimental arm did not show meaningful changes in the enzymohistochemical test despite having been treated with ME-344 (Figure 3B). Similarly, we did not find meaningful changes between the pre- and post- treatment transcriptional levels of either of the SDH units, regardless of the treatment arm or experiencing or not vascular normalization (Figure 3B). Although the preclinical data suggest that the test translates truly mitochondrial respiration, and is sensitive to pharmacological blockade, the lack of meaningful results in the clinical setting may be explained by several reasons: first, the SDH-EHC is a very sensitive technique that requires fresh tissue to measure mitochondrial activity *in vivo*. Therefore, the longer the time between the tumor biopsy and its correct preservation may impact on the quality of the tissue that start degradation and become hypoxic. Within the context of a multicentric randomized clinical trial, it is challenging to obtain snap-frozen tumor tissue within a <5-minutes time-window in a consistent manner across all centers. For that reason, it is biologically plausible that the role of SDH-EHC as a biomarker of ME-344 activity remains preliminary and requires optimization. Another potential option is timing: whereas in animals daily dosage is feasible and tissue can be obtained at any moment after drug administration, patients had their post-treatment biopsy harvested a minimum of seven days after the last ME-344 dose. At this moment, the effect over complex I could be already absent given the ME-344 pharmacokinetic properties [32]. Thus, in order to optimize the use of mitochondrial inhibitors, on top of searching for the right “metabolic context”, the search for potential pharmacodynamic markers should continue.

### Further implications of metabolic adaptation against antiangiogenics: breast cancer immuno-oncology

In the era of immuno-oncology (IO), a mention to the potential implications of any experimental or standard drug for enhancing the activity of IO drugs is mandatory. In the context of breast cancer, where IO drugs, albeit active, have achieved lower efficacy rates than those observed in melanoma or lung cancer [47-49], this is particularly relevant. Antiangiogenics have the potential to modulate the tumor microenvironment by correcting – or increasing – hypoxia. Vascular normalization results in a more homogeneous distribution of functional tumor vessels facilitating CD4+ and CD8+ T-cell tumor infiltration [50]. The presence of tumor-infiltrating lymphocytes (TILs) have been consistently associated with a more-favourable prognosis and better outcome [51, 52]. On the other hand, abnormal tumor vasculature fosters an immunosuppressive tumor microenvironment that enables the tumor to evade host immunosurveillance. Tumor hypoxia upregulates the transcription factor HIF1α that favours the recruitment of regulatory T-cells (T_regs_) and increases the expression of PD-L1 in tumor cells [53]. Interestingly, in the BR-009 trial, we observed a reduction in the percentage of FOXP3 T_regs_ positive cells in tumors from those patients that experienced bevacizumab-induced vascular normalization compared with those patients with increased abnormality of vasculature, regardless of having been treated in the standard or experimental arm (Figure 3C). However, and intriguingly, we also found a higher frequency of FOXP3+-positive cells in ME-344 treated patients compared with placebo. The metabolic properties of T_regs_ *in vivo* are still a highly controversial topic, although the role of mitochondria-driven oxidative phosphorylation is demonstrated to be critical for T_regs_ functionality [54]. It could be expected that the inhibition of complex I of respiratory chain by ME-344 would affect the viability or functionality of FOXP3+ T_regs_ cells. However, it has been shown as well that T_regs_ are able to rely on lipid metabolism to thrive in low glucose environments, suggesting that lipid metabolism might be responsible for their survival within tumors [55, 56]. Moreover, it was nicely demonstrated in mice that T_regs_ cell-specific ablation of mitochondrial respiratory chain complex III displayed a loss of the cell suppressive capacity of T_regs_ without altering its cell proliferation and survival [57]. The results of the BR-009 trial in this regard may be translating the resulting effect of vascular normalization on one hand, and mitochondrial inhibition on the other; in addition, the results shown in Figure 3C are merely morphological and not functional (i.e., they translate the “amount” of FOXP3+-positive T_regs_ cells, but they do not inform about their tolerogenic or antitumoral activity). Nevertheless, further preclinical work should clarify the context (if and in which patients) of potential use of IO drugs (such as anti-CTLA4 or anti-PD-1/L1 monoclonal antibodies) in combination with antiangiogenic plus antimitochondrial combos.

### Lesson learned and future research lines

Taken together, our results seem to confirm that the activity of antimitochondrial agents is dependent on the metabolic context, and that upon vascular normalization, a context for metabolic synthetic lethality is generated. In addition, our work highlights the need for tracking vascular normalization in order to guide the efficacy of antiangiogenic drugs: whereas the same agent can induce normalization in a patient and increase the abnormality in one another with the same tumor type, and considering that each antiangiogenic agent seems to display different normalizing properties, monitoring the glucose and/or misonidazole uptake seems to inform reasonably well who is experiencing one or the other outcome in real time. This is of key importance, since patients experiencing normalization seem to get more benefit in general from standard chemotherapy combinations, and also could be amenable for combination with antimitochondrial agents. Conversely, patients showing increased abnormality could be offered alternative treatments. However, several obstacles are still needed in order to adopt such strategies in the clinical setting. First, the ratio of vascular normalization, which clearly does not occur in a homogeneous manner across all patients, should be addressed prospectively for the most common antiangiogenics in tumors for which there is currently an approved FDA-indication. A double approach, including a dynamic imaging test plus correlative immunohistochemical studies should be followed. In addition, dynamic imaging tests involving radiotracers such as [18F]-FMISO-PET and / or FDG-PET should be standardized. Practical issues such as establishing a clear cut-off value for hypoxia or normoxia, and minimum values of tracer uptake change along treatment for establishing whether a patient is experiencing truly normalization or just minor changes should be clarified. The timing for longitudinal re-assessment should be established as well for each agent (i.e., not all agents may induce normalization or abnormality within the same timeframe, due to very different half-lives and pharmacologic properties of different antiangiogenics). In order to proceed with larger trials seeking for metabolic synthetic lethality in combination with antimitochondrial agents, the former two conditions should be fulfilled. In case this is not achieved, only antiangiogenic agents with high normalization ratio (i.e., at least 50% of the treated patients, as opposed for example of just 1/3 of the patients in the BR009 trial) should be combined with antimitochondrial agents, in order to maximize the number of patients experiencing the desired effect. Furthermore, more research is needed in the field of pharmacodynamic markers of activity for mitochondrial inhibitors. Clearly, our approach based on a highly sensitive EHC test did not work, since it did not consider the logistic complexity of a randomized clinical trial for tissue preservation. More robust markers, less sensitive to timing to tissue storage or tissue processing should be explored. Once this is defined, only agents showing unequivocal pharmacodynamic engagement should move forward in clinical development.

From a broader perspective, since so far and at least in breast cancer mitochondrial inhibitors seem to have restricted their activity in the context of antiangiogenic therapy, a general solution has to be given to the escape to this drug class. It seems clear now that patients that experience normalization can have benefit from mitochondrial or other metabolic inhibitors. However, further research is required to understand which is the escape mechanisms in tumors experiencing increased vascular abnormality, and if such mechanism is therapeutically targetable. Once this conundrum is solved, we propose the trial depicted in Figure 3D in order to exploit the advantages of targeting escape mechanisms no matter which of the two possible phenomena is observed upon exposure to antiangiogenics. Finally, in the era of immune-oncology it is required to decipher to what extent the changes induced by vascular normalization, at the stromal, structural, and metabolic level, alone and in combination with mitochondrial inhibitors, influence positively or negatively the function of different immune cell subpopulations, making the tumors more or less susceptible to benefit from IO drugs. Whichever is the case, we are at the dawn of an exciting era of rationale personalized multi-targeted combos guided by accurate biomarkers that will incorporate many concepts of basic science in the routine clinical care of oncology.
